# Detection of Diverse N-Acyl-Homoserine Lactones in *Vibrio alginolyticus* and Regulation of Biofilm Formation by N-(3-Oxodecanoyl) Homoserine Lactone *In vitro*

**DOI:** 10.3389/fmicb.2017.01097

**Published:** 2017-06-16

**Authors:** Jianfei Liu, Kaifei Fu, Yuxiao Wang, Chenglin Wu, Fei Li, Lei Shi, Yinlin Ge, Lijun Zhou

**Affiliations:** ^1^Department of Biochemistry and Molecular Biology, Medical College of Qingdao UniversityQingdao, China; ^2^Central Laboratory, Navy General Hospital of PLABeijing, China; ^3^Institute of Food Safety and Nutrition, Jinan UniversityGuangzhou, China

**Keywords:** N-acyl homoserine lactones, quorum sensing, *Vibrio alginolyticus*, 3-oxo-C_10_-HSL, biofilm formation, biofilm matrix, CLSM

## Abstract

Quorum sensing (QS) is a cell-to-cell communication system based on the exchange of small intercellular signal molecules, such as N-Acyl homoserine lactones (AHLs), which act as cell-density mediators of QS gene expression, and are highly variable both in types and amounts in most Gram-negative Proteobacteria. Understanding the regulation of AHLs may contribute to the elucidation of cell density-dependent phenomena, such as biofilm formation. *Vibrio alginolyticus* is among the most frequently observed marine opportunistic *Vibrio* pathogens. However, AHL production of this species and its effects on biofilm formation remain to be understood. Here, our study reported the diverse AHL profiles of 47 marine-isolated *V. alginolyticus* strains and the effects of exogenous 3-oxo-C_10_-HSL on biofilm formation under different temperature conditions (16°C and 28°C). A total of 11 detected AHLs were produced by the isolates, of which 3-OH-C_4_-HSL, 3-oxo-C_10_-HSL and 3-oxo-C_14_-HSL comprised the largest proportions. We also observed that moderate levels of exogenous 3-oxo-C_10_-HSL (10 and 20 μM) could induce or enhance biofilm formation and alter its structure, while high levels (40 and 100 μM) did not significantly improve and even inhibited biofilm formation in *V. alginolyticus*. Further, regulation by exogenous 3-oxo-C_10_-HSL was both concentration- and temperature-dependent in *V. alginolyticus*.

## Introduction

Quorum Sensing (QS) is an important communication system used by bacterial cells, which allows monitoring of cell density and regulation of functions within the population. This process depends on the production, secretion, accumulation and recognition of signaling autoinducers (AIs; Bassler, 1999). The initial regulation of QS provides a cascade of controls that propels the bacterial community to express an advantageous phenotype and ensure their survival (Williams et al., 2007; Hawver et al., 2016). Further studies revealed that QS could also receive feedback from its components, allowing the cells to adjust their regulation in real-time (Reuter et al., 2016). Efficiency in changing the bacterial phenotype controlled by QS during proliferation is a key factor to effectively coordinate the behavior of the entire bacterial population, such as the biofilm formation in response to hostile conditions, thus, enhancing the survival of Proteobacteria.

N-acyl homoserine lactones (AHLs) are a typical group of small AI molecules that mediate the QS phenomenon in gram-negative Proteobacteria especially in responding to changes in the environment (Williams et al., 2007; Garcia-Aljaro et al., 2008). AHLs are amphiphilic chemical compounds that share the same structure including a hydrophilic homoserine lactone ring and a hydrophobic acyl side chain (O'Connor et al., 2015). The diversity of AHLs is based on the number of carbon atoms (4, 6, 7, 8, 10, 12, 14, 16, or 18) on the acyl side chain and substituent (hydrogen, oxhydryl or carbonyl; Kumari et al., 2006) present on the 3rd carbon atom, which are also the basis for distinction and important in regulating specificity. As an essential part of the QS system, AHL molecules are synthesized by certain synthases (e.g., LuxI type synthases) and bind to transcripts (e.g., LuxR type regulators), followed by the binding of the AHL-receptor complex to DNA, initiating the downstream regulation of QS-controlled genes (Swift et al., 2001). The N-(β-ketocaproyl)-homoserine lactone (3-oxo-C_6_-HSL) in *Vibrio fischeri* was the first AHL to be described (Eberhard et al., 1981), and subsequent studies have uncovered more AHLs produced by other Gram-negative Proteobacteria. These include N-butyryl-homoserine lactone (C_4_-HSL), N-(3-hydroxybutyryl)-homoserine lactone (3-OH-C_4_-HSL), N-hexanoyl-homoserine lactone (C_6_-HSL), N-(3-oxodecanoyl)-homoserine lactone (3-oxo-C_10_-HSL), among others (Valiente et al., 2009; Wang et al., 2013; Tan W. S. et al., 2014; Jamuna and Ravishankar, 2016). AHLs are vital in the capacity of pathogenic Proteobacteria to invade surfaces, and are also involved in multiple physiological processes such as bioluminescence, production of virulence factors, biofilm formation and drug resistance (Horng et al., 2002; Lumjiaktase et al., 2006; Garcia-Aljaro et al., 2012) as summarized in Table 1.

**Table 1:** Biological and physiological functions of AHL molecules.

**AHL molecules**	**Biological and physiological functions**	**Bacteria/host**	**References**
C_4_-HSL	Tissue infection; biofilm formation	*Vibrio vulnificus*; *Vibrio sinaloensis*	Valiente et al., 2009; Tan P. W. et al., 2014
C_6_-HSL	Biofouling; bacterial cells attachment; multidrug resistance; carpospore liberation; pili assembly	*Bosea massiliensis*; *Escherichia coli*; *Bacteroides fragilis*; *Shewanella algae*; *Acinetobacter baumannii*	Pumbwe et al., 2008; Luo et al., 2015; Okutsu et al., 2015; Singh et al., 2015; Jamuna and Ravishankar, 2016
3-OH-C_6_-HSL	Phenazine production	*Pseudomonas chlororaphis* subsp. *aurantiaca*	Morohoshi et al., 2017
3-oxo-C_6_-HSL	Bioluminescence; pigmentation; prodigiosin production; biofouling	*V. fischeri*; *Serratia marcescens*; *B. massiliensis*	Eberhard et al., 1981; Horng et al., 2002; Okutsu et al., 2015
C_8_-HSL	Sliding motility; biofilm formation; oxidative stress protection	*S. marcescens*; *P. aeruginosa*; *Burkholderia pseudomallei*	Horng et al., 2002; Lumjiaktase et al., 2006; Abbas et al., 2007
3-oxo-C_8_-HSL	Violacein production; biofouling	*Pseudoalteromonas ulvae*; *B. massiliensis*	Mireille Aye et al., 2015; Okutsu et al., 2015
C_10_-HSL	Biofouling; extracellular hydrolytic enzymes production; plant virulence and pathogenicity; biofilm formation; primary root length inhibition	*Lysobacter* sp.; *Pantoea ananatis*; *Pseudomonas fuscovaginae*; *Serratia liquefaciens*; *Aeromonas sobria*; *Arabidopsis thaliana* (host)	Mattiuzzo et al., 2011; Zhao et al., 2014; Jatt et al., 2015; Okutsu et al., 2015; Zhang et al., 2016
3-oxo-C_10_-HSL	Mupirocin resistance; biofouling; extracellular protease activation; hemolysin activation	*Pseudomonas fluorescens*; *Lysobacter* sp.; *Vibrio fluvialis*	El-Sayed et al., 2001; Wang et al., 2013; Okutsu et al., 2015
C_12_-HSL	Biofilm formation; plant virulence factor activation; bacterial pathogenicity	*S. enteritidis*; *P. fuscovaginae*	Mattiuzzo et al., 2011; Campos-Galvao et al., 2016
3-OH-C_12_-HSL	Biofilm formation; bacterial pathogenicity	*Vibrio scophthalmi*	Garcia-Aljaro et al., 2012
3-oxo-C_12_-HSL	Inflammation; immune response activation; host immune response activation; swimming and adhesion inhibition; biofilm formation	*P. aeruginosa*; *P. ulvae*; *A. hydrophila*	Khajanchi et al., 2009; Vikstrom et al., 2009; Davis et al., 2010; Mireille Aye et al., 2015
3-oxo-C_14_-HSL	Callose deposition; accumulation of phenolic compounds; lignification of cell walls enhancement; modulation of membrane dipole potential	*P. aeruginosa*; *Arabidopsis thaliana* (host)	Davis et al., 2010; Schenk et al., 2014
3-OH-C_14_-HSL	Plant pigmentation	*Rhodospirillum rubrum*	Mastroleo et al., 2013

Biofilm formation is an important characteristic of bacterial communities that enhances invasion leading to infection, drug resistance, and pathogenicity (Soto et al., 2007; Naves et al., 2008). Thus, it is critical to elucidate the underlying mechanisms involved in QS system to further understand biofilm formation. Recent studies showed that QS controls the transformation of bacteria from being free-living or planktonic to colonial or biofilm-forming state, and further regulates and coordinates the behavior of the entire community, allowing synchronized response to environmental challenges to enhance viability (Mireille Aye et al., 2015; Okutsu et al., 2015). QS further regulates the construction of biofilm matrix and speeds up the process of biofilm formation (Tseng et al., 2016). It could also indirectly upregulate biofilm thickness by increasing bacterial motility (Yang et al., 2014). QS also increases dispersal of detached bacteria from the matured biofilm to trigger a new developmental cycle of biofilm formation (Emerenini et al., 2015).

Increasing reports of characterized AHLs in different bacterial species have provided valuable insights into how AHLs regulate bacterial biofilm formation. For example, Vinoj et al. (2014) showed that AHLs produced by *Vibrio parahaemolyticus* could regulate formation of biofilm and enhancement of colonization. Meanwhile, N-octanoyl-homoserine lactone (C_8_-HSL) and 3-oxo-C_12_-HSL were also found to be responsible for biofilm formation in *Pseudomonas aeruginosa* and *Aeromonas hydrophila*, respectively (Abbas et al., 2007; Khajanchi et al., 2009). N-dodecanoyl-homoserine lactone (C_12_-HSL) positively regulated biofilm formation in *Salmonella enteritidis* (Campos-Galvao et al., 2016). Huang et al. (2009) further showed that AHLs changed in the course of biofilm formation, first being dominated by short side-chain AHLs followed by an increase in long side-chain AHLs, indicating a feedback regulation mechanism. However, bacterial growth, QS molecules and biofilm formation could also be inhibited by key abiotic variables, such as temperature and composition of the culture medium (Yates et al., 2002; Sheng et al., 2013; Turner et al., 2014; Lamas et al., 2016). Hare et al. (1981) for example, observed that the production of extracellular collagenase and alkaline protease needed for biofilm formation were inhibited when the temperature increased from 30°C to 37°C.

*Vibrio alginolyticus* is one of the most abundant aquatic pathogenic *Vibrio* (Mechri et al., 2013), proliferating well in a wide range of environments including offshore and coastal areas, rivers, sediments, and saline waters (Narracci et al., 2014). It also has a wide geographic distribution, with recorded presence in several marine environments, such as east (He et al., 2016) and south China Sea (Wu et al., 2016), west Korea sea (Kang et al., 2016), and Indian Ocean (Gauzere et al., 2016). This environmental opportunistic pathogen has long been a threat to fishing industry, and has been reported to cause human diseases worldwide. Human *V. alginolyticus* infections include several acute and even deadly conditions like diarrhea, septicemia, and the inflammation of multiple tissues (Caccamese and Rastegar, 1999; Sganga et al., 2009; Gauzere et al., 2016). So far, there are relatively fewer studies on biofilms of *V. alginolyticus*, and mostly concentrated on virulence-related genes in correlation with biofilm formation. Despite evidence that biofilm forming *V. alginolyticus* strains activate stronger immune response in juvenile tiger shrimp than planktonic strains, and that the former were superior to the latter in stimulating non-specific immune response (Sharma et al., 2010), studies on biofilm effects in human infection remain limited.

Recent studies on genetic basis of QS regulation in *V. alginolyticus* mostly focused on the possible effects of LuxR type genes (i.e., virulence related gene Hfq) and QS signaling transcriptional regulators, e.g., motility regulated extracellular protein Pep and the colony phenotype intermediated protein valR (Chang et al., 2010; Cao et al., 2011; Liu et al., 2011). Although the LuxR type homolog of *V. alginolyticus* could induce the alteration of colony phenotype and regulate flagellar biosynthesis relating to its biofilm formation (Chang et al., 2010), the direct QS signaling control on *V. alginolyticus* biofilm formation has not yet been well explored, which might be partly attributed to the less understood production of AHLs in *V. alginolyticus*. To our knowledge, no detailed profiling of AHL signals and their effects on biofilm formation have yet been carried out in *V. alginolyticus*. Therefore, the elucidation of AHL profiles in *V. alginolyticus* and their relationship with biofilm formation is of interest to the control of *V. alginolyticus* infections.

To fill in some of these knowledge gaps, with the aim of gaining further understanding of mechanisms involved in QS, our study focused on the identification of different AHLs in *V. alginolyticus* strains, which were also used to investigate the detailed relationship between biofilm formation and AHLs under different temperature conditions. The acquired knowledge provides interesting perspectives regarding the roles of QS signaling molecules in aquatic pathogens.

## Materials and methods

### Bacterial isolation and growth conditions

A total of 47 strains of marine *V. alginolyticus* (hereafter referred to as strains N°01–N°47) were isolated from Bohai, China and cultured in 2216E broth (BD Biosciences, USA) at 28°C and 180 rpm of shaking. Biochemical identification was performed using the VITEK 2 compact system (Biomérieux, France), following the manufacturer's instructions. For the cross-feeding assay, the transformed biosensor strain *Chromobacterium violaceum* CV026 was used for short side-chain AHLs detection (C_4_-HSL–C_8_-HSL; McClean et al., 1997; Ravn et al., 2001), and was cultured in Luria Bertani (LB) broth with 40 μg/mL kanamycin (Sigma, USA) at 28°C and 180 rpm of shaking for 16 h. The transformed biosensor strain *Agrobacterium tumefaciens* KYC55 (JZA1-1) was used for long side-chain AHLs (C_8_-HSL–C_14_-HSL) detection (Zhu and Mekalanos, 2003; Golberg et al., 2011), and was cultured in LB broth with 1 μg/mL tetracycline, 100 μg/mL spectinomycin and 100 μg/mL gentamycin (Sigma, USA) at 28°C and 180 rpm of shaking for 16 h. The positive control strains *Erwinia carotovora* GS101 (Chhabra et al., 1993) and *P. aeruginosa* PAO1 (Tateda et al., 2003) were cultured in LB broth for 24 h and 16 h, respectively. All bacterial strains used in this study were listed in Supplementary Table 1.

### AHL detection of *V. alginolyticus*

#### Cross-feeding assay for AHL production

To determine the range of AHLs produced by strains N°01–N°47, the bacterial suspension was cross-fed with 2 biosensor strains (*C. violaceum* CV026 and *A. tumefaciens* KYC55) following the methods reported by Han-Jen et al. (2013) with modifications. Briefly, strains were cultured for 36 h and pelleted by centrifugation at 10,000 rpm for 15 min. Bacterial suspension was adjusted to a concentration of approximately 5 × 10^6^ Colony Forming Units per milliliter (CFU/mL; OD_570_ = 0.03), incubated with the reporter strains for 36 h at 28°C. Before using *A. tumefaciens* KYC55 as the reporter strain, a new layer of 5-bromo-4-chloro-3-indolyl-β-D-galactoside (X-Gal, Amresco, USA) was added on the agar plate.

#### AHLs profiling through high-performance liquid chromatography tandem mass spectrometry (HPLC-MS/MS) assay

The HPLC-MS/MS method was used to quantify the AHLs produced by all *V. alginolyticus* strains (N°01–N°47). The 47 strains were adjusted to approximately 3 × 10^5^ CFU/mL and were cultured for 36 h. After incubation, each culture was centrifuged at 10,000 rpm for 10 min and AHLs in supernatants were extracted with ethyl acetate with 0.1% (v/v) formic acid. Extracts were freeze-dried using an ALPHA 1-2LD plus lyophiliser (CHRIST, Germany) as previously described in Tan W. S. et al. (2014). The extract was resuspended in 80 μL of 99.9% HPLC-grade methanol (Thermo Fisher, USA) and was analyzed by HPLC-MS/MS.

The Prominence UFLC-XR (SHIMADZU, Japan) system was utilized for HPLC analysis with a Symmetry C_18_ reverse-phase column (3.5 μm, 2.1 × 100 mm; Waters, USA). Subsequently, MS analysis was performed on a Q-trap 5500 (AB SCIEX, USA) system using MRM mode with positive ion scanning. C_4_-HSL, N-(3-hydroxybutyryl)-homoserine lactone (3-OH-C_4_-HSL), C_6_-HSL, 3-oxo-C_6_-HSL, C_8_-HSL, N-(3-hydroxyoctanoyl)-homoserine lactone (3-OH-C_8_-HSL), N-(3-oxooctanoyl)-homoserine lactone (3-oxo-C_8_-HSL), N-decanoyl-homoserine lactone (C_10_-HSL), 3-oxo-C_10_-HSL, C_12_-HSL, N-(3-hydroxydodecanoyl)-homoserine lactone (3-OH-C_12_-HSL), 3-oxo-C_12_-HSL, N-(3-hydroxytetradecanoyl)-homoserine lactone (3-OH-C_14_-HSL), and N-(3-oxotetradecanoyl)-homoserine lactone (3-oxo-C_14_-HSL) were selected as the standards purchased from Sigma (USA). Two mmol/L ammonium acetate and 0.1% (v/v) formic acid were diluted in water as mobile phase A or in methanol as mobile phase B. The flow rate (0.2 mL/min), analysis time (40 min/sample), and mobile gradient profile were optimized (Supplementary Table 2), and 20 μL of each sample was analyzed. The following conditions were used for MS: electron spray ionization (ESI) was set at 4.5 kV, the curtain gas (CUR) at 20 Psi, collision gas (CAD) at medium, temperature (TEM) at 650°C, ion source gas 1 (GS1) at 40 Psi, and ion source gas 2 (GS2) was set at 45 Psi. Also, optimum quantitative ion pairs (*m/z*) were determined under Multiple Reaction Monitoring (MRM) mode following McLafferty and Turecek (1993) rearrangement (Supplementary Table 3), with a linear correlation coefficient greater than 0.99 for each standard compound (Supplementary Table 4). Standard curves were drawn with the peak area on the *Y*-axis and the corresponding concentration on the *X*-axis from which the slope was calculated.

### Detection of *V. alginolyticus* biofilms

#### Biofilm formation

The 47 strains (adjusted to 3 × 10^5^ CFU/mL) were incubated in 96-well polystyrene microplates (Corning, USA) at 28°C for 36 h from which biofilm formation was monitored. A semi-quantitative adhesion test modified from Stepanovic et al. (2000) was used. The CFU of suspended cultures were counted. The microplates were first rinsed with phosphate-buffered saline (PBS) solution and fixed with Bouin's fluid (LEAGENE, China) for 20 min, and then stained with crystal violet (0.1%, w/v solution; LEAGENE, China) for 30 min, after which the excess crystal violet was washed off. The biofilms were dissolved in 95% (v/v) ethanol and quantified at OD_570_ with a microplate reader (Model 680, BIO-RAD, USA). The standardized biofilm (BF) was calculated using the following formula: Standardized OD_570, sample_ = (original OD_570, sample_ − OD_570, control_)/log_CFU/mL_).

Effect of temperature on biofilm formation was then investigated by subjecting the strains to a gradient of temperature. Temperature regimes used were based on those previously described by De Oliveira et al. (2014) and Miller et al. (2016). Specifically, 3 temperatures (16, 28, and 40°C) were selected, hereafter referred to as low, moderate and high temperature conditions, respectively. Strain N°24 (no 3-oxo-C_10_-HSL production and weak biofilm formation) and strain N°40 (high level of 3-oxo-C_10_-HSL production and strong biofilm formation) were selected from the 47 strains for this particular part of the study. Cultures were adjusted to 3 × 10^5^ CFU/mL before being used.

To explore the effects of exogenous AHLs, specifically 3-oxo-C_10_-HSL, on biofilm formation under different temperature conditions, strains N°24 and N°40 were separately supplemented with exogenous 3-oxo-C_10_-HSL at final concentrations of 1, 2, 5, 10, 20, 40, or 100 μmol/L. For biofilm formation detection, 200 μL of 3-oxo-C_10_-HSL treated strains were incubated in 96-well polystyrene microplates at 16°C or 28°C for 36 h, and a culture prepared with DMSO (AppliChem, Germany) was used as the negative control. Finally, 1 mL of these cultures were incubated in a two-chamber cell imaging cover glass system (Eppendorf, Germany) while inclined to approximately 45° to form a clear liquid-air interface, and was placed in a moist sterile incubation box for 36 h at 16°C or 28°C before being used for confocal laser scanning microscopy (CLSM) imaging. All treatments and assays were performed in triplicates.

### Fluorescence labeling microscopy (FLM) assay

Strains N°24 and N°40 were incubated in a 4-well glass Lab-Tek®II Chamber Slide System (NUNC, Denmark) while inclined at approximately 45° to form a clear liquid-air interface, and placed in a moist sterile incubation box. The FLM assay was performed in the same cultures after 12, 24, 36, 48, 60, 72, 84, 96, 108, and 120 h incubation period. Treatments for each strain were in triplicates. The wells were rinsed with PBS and fixed with 4% paraformaldehyde (LEAGENE, China) for 30 min. Then, the wells were labeled by FITC-ConA (Sigma, USA) for exopolysaccharides (EPS) and propidium iodide (PI; Sigma, USA) for bacterial nucleic acid, before rinsed finally with PBS. The slide was sealed with antifade mounting medium (Beyotime, China).

The pictures were taken with a Nikon ECLIPSE Ti-S Inverted Fluorescence Microscope (Nikon, Japan) equipped with 33 mm ND4/ND8 filters employing green filter detecting PI fluorescence (500–550/615 nm excitation/emission wavelengths) and blue filter to detect FITC fluorescence (400–490/525 nm excitation/emission wavelengths). The pictures were processed with NIS-Elements BR 3.0 software (Nikon, Japan).

#### CLSM assay of the biofilm matrix

The biofilms were treated in the same manner as described in Section CLSM Assay of the Biofilm Matrix. Z-scans of the images were taken using LSM710 3-channel Zeiss confocal laser scanning microscope (Zeiss, Germany) equipped with TwinGate main beamsplitter employing 543/576–718 nm and 488/493–542 nm excitation/emission wavelengths. Scans were processed and reconstructed into 3D images using Zen v. 2.3 (Zeiss, Germany).

Five specific morphological traits were used as indices of biofilm structure as obtained from CLSM images, namely biomass, average and maximum thickness, roughness coefficient, and microcolonies at the substrate (Derlon et al., 2010), which were quantified and analyzed using the COMSTAT 2.1 software following Heydorn et al. (2000) and Vorregaard (2008).

Analysis of variance (ANOVA) and detection of significant differences (Dunnett's test) were carried out using the standardized OD_570_ data in SPSS 19 (IBM Statistics, USA). All *P*-values were two-tailed, and the threshold for statistical significance was set at 0.05. All results were presented as the mean values ± standard deviations (SD) for all independent experiments in each group.

## Results

### AHL profiling of *V. alginolyticus*

**Figure 1:**
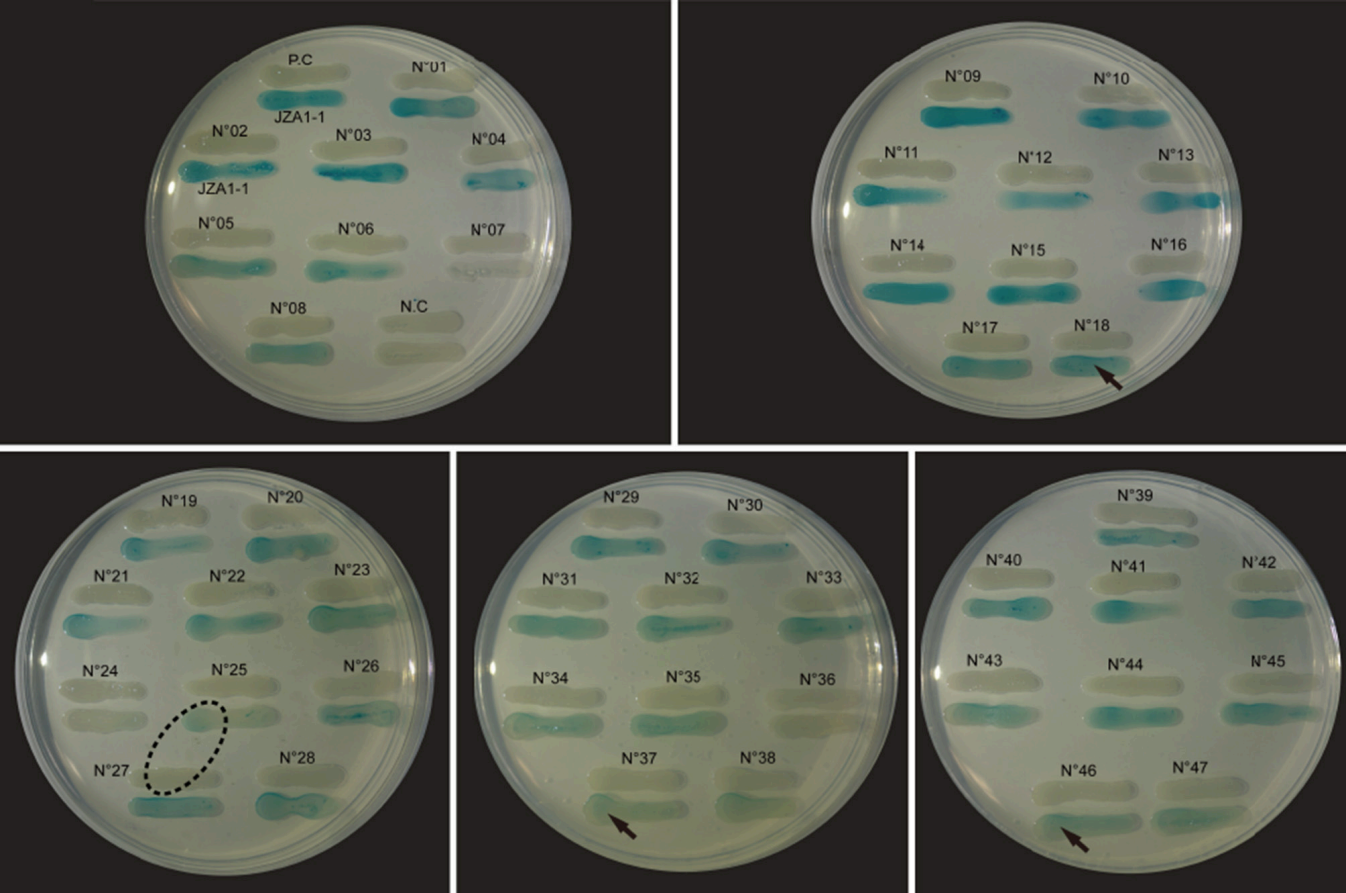
Detection of long side-chain AHL production in *V. alginolyticus*. Strain suspensions cross fed with biosensor *A. tumefaciens* KYC55. The “JZA1-1” represents biosensor *A. tumefaciens* KYC55; the “P.C” (positive control) represents *P. aeruginosa* PAO1; the “N.C” (negative control) represents *A. tumefaciens* KYC55; the “arrow” symbol represents the visible color change induced from the proximal end to the distal end of the inducer; the “dashed oval” symbol represents the surface diffusion of the color change from the adjacent inducer.

Cross-feeding results showed that no bacterial suspension induced visible violacein production in *C. violaceum* CV026, indicating a lack of short side-chain AHL production by the tested *V. alginolyticus* strains. However, 43 out of the 47 strain suspensions (Figure 1) showed distinct blue color change in *A. tumefaciens* KYC55 with varying color intensities. In addition, traces of diffusing blue color on reporter strain was observed next to strain N°25, which could indicate a strong long side-chain AHL production by adjacent strain N°27. The bioassay results confirmed that most of the *V. alginolyticus* strains we tested produced long side-chain AHLs.

HPLC-MS/MS results further showed that the *V. alginolyticus* strains produced a total of 11 different AHLs, with each strain producing more than 6 AHL types. Table 2 lists all the detailed information about the detected AHLs and concentration classification of 47 strains, the results showed that the concentrations of short side-chain AHLs were all very low except for 3-OH-C_4_-HSL, while those of the long side-chain AHLs were in a large scale and varied in between strains. The AHLs 3-oxo-C_10_-HSL, 3-OH-C_4_-HSL, and 3-oxo-C_14_-HSL were the most dominant and were produced by all the tested strains except for strain N°24 (no 3-oxo-C_10_-HSL production). C_6_-HSL was detected only in trace concentrations and only produced by strain N°03. Lastly, 3 AHLs including C_8_-HSL, 3-oxo-C_8_-HSL and 3-OH-C_14_-HSL were not detected in all tested strains.

**Table 2:** AHL profiling of the 47 *V. alginolyticus* strains by HPLC-MS/MS.

**Strain number**	**AHLs concentration level**														**Detectable AHL numbers**
	**C_4_**	**3-OH-C_4_**	**C_6_**	**3-oxo-C_6_**	**C_8_**	**3-OH-C_8_**	**3-oxo-C_8_**	**C_10_**	**3-oxo-C_10_**	**C_12_**	**3-OH-C_12_**	**3-oxo-C_12_**	**3-OH-C_14_**	**3-oxo-C_14_**	
N°01	+	++++	−	+	−	+	−	++	+++	++	+	++	−	+++++	10
N°02	+	+++	−	+	−	+	−	++	+++	++	+	+	−	+++++	10
N°03	+	+++++	+	+	−	+	−	++	+++++	++	++	++	−	++++	12
N°04	+	+++	−	+	−	+	−	+	+	−	+	+	−	+++	9
N°05	+	++++	−	+	−	+	−	++	+++	−	+	+	−	+++	9
N°06	+	+++	−	+	−	+	−	+	+++	−	+	+	−	+++	9
N°07	−	+++++	−	+	−	+	−	+++	++	−	−	+	−	++	7
N°08	+	+++	−	+	−	+	−	++	+++	++	+	+	−	+++	10
N°09	−	+++++	−	+	−	+	−	++	+++++	++	++	++	−	+++	9
N°10	+	+++	−	+	−	+	−	++	+++	++	+	+	−	+++++	10
N°11	+	++++	−	+		+		++	++++	+	++	++		++++	11
N°12	+	+++	−	+	−	+	−	++	++	++	+	+	−	+++	10
N°13	+	+++	−	+	−	+	−	++	+++	−	+	+	−	+++	9
N°14	+	++++	−	+	−	+	−	++	+++++	+	++	+	−	++++	10
N°15	+	+++	−	+	−	+	−	+	+++++	++	+	+	−	++++	10
N°16	+	+++	−	+	−	+	−	++	+++	−	+	++	−	+++	9
N°17	+	+++	−	+	−	+	−	++	+++	+	+	+	−	+++	10
N°18	+	++++	−	+	−	+	−	++	+++	−	+	+	−	+++	9
N°19	+	+++	−	+	−	+	−	++	+++	−	+	++	−	++++	9
N°20	+	+++	−	+	−	+	−	+	++	++	+	+	−	++++	10
N°21	+	+++	−	+	−	+	−	++	+++	−	+	++	−	++++	9
N°22	+	++++	−	+	−	+	−	++	++	−	+	++	−	++++	9
N°23	+	++++	−	+	−	+	−	++	+++	−	+	+	−	++++	9
N°24	−	+++++	−	+	−	+	−	++	−	−	−	+	−	+++	6
N°25	+	++++	−	+	−	+	−	++	+++	++	+	+	−	+++	10
N°26	−	+++	−	−	−	+	−	++	+++	+	+	+	−	++++	10
N°27	+	+++	−	−	−	+	−	++	+++	+	+	+	−	++++	10
N°28	+	++++	−	+	−	+	−	++	+++	−	+	++	−	++++	9
N°29	+	++++	−	+	−	+	−	++	+++	++	+	++	−	++++	10
N°30	+	++++	−	+	−	+	−	+	++++	−	+	++	−	+++	9
N°31	+	+++	−	+	−	+	−	++	+++	−	+	+	−	++++	9
N°32	+	+++	−	+	−	+	−	++	+++	+	+	+	−	++++	10
N°33	+	++++	−	+	−	+	−	+++	+++	−	+	+	−	++++	9
N°34	+	+++	−	+	−	+	−	++	+++	−	+	++	−	++++	9
N°35	−	++++	−	+	−	+	−	+++	+++	−	−	++	−	++++	7
N°36	−	++++	−	+	−	+	−	++	+++	+	++	+	−	+	9
N°37	+	+++	−	+	−	+	−	+	+++	++	+	+	−	+++	10
N°38	+	+++	−	+	−	+	−	++	+++	−	+	++	−	+++	9
N°39	+	++++	−	+	−	+	−	++	+++	++	++	+	−	++++	11
N°40	+	++++	−	+	−	+	−	+++	+++++	−	+	++	−	++++	9
N°41	+	+++	−	+	−	+	−	++	+++	−	+	++	−	+++++	9
N°42	+	+++	−	+	−	+	−	++	+++	++	+	+	−	++++	10
N°43	+	+++	−	+	−	+	−	++	+++	++	+	+	−	++++	10
N°44	+	+++	−	+	−	+	−	++	+++	+	+	+	−	++++	10
N°45	+	++++	−	+	−	+	−	+++	+++	−	+	+	−	++++	9
N°46	+	+++	−	+	−	+	−	++	+++	++	+	+	−	++++	10
N°47	+	++++	−	+	−	+	−	++	++++	+	++	+	−	+++	10

*AHL levels produced by the marine-isolated V. alginolyticus strains were calculated and classified according to their concentrations. The “−” represents for not detected; the “+” represents that AHL concentrations < 10 nmol/L; the “++” represents that 10 nmol/L < AHL concentrations < 100 nmol/L; the “+++” represents that 100 nmol/L < AHL concentrations < 1 μmol/L; the “++++” represents that 1 μmol/L < AHL concentrations <5 μmol/L; the “+++++” represents that AHL concentrations > 5 μmol/L*.

### Biofilms formed by *V. alginolyticus* strains

**Figure 2:**
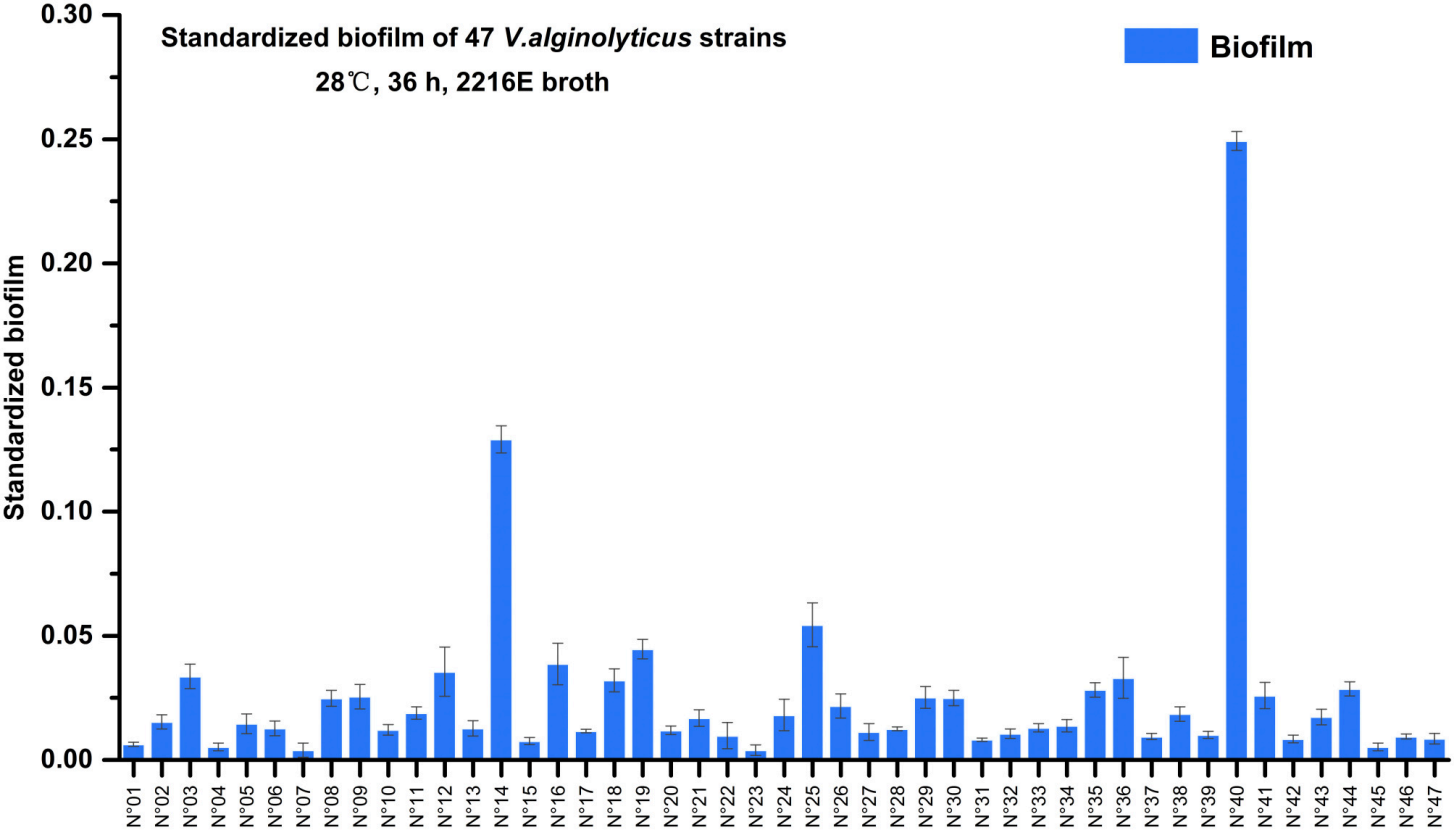
Biofilms formation of 47 marine-isolated *V. alginolyticus* strains. The ability of 47 marine-isolated *V. alginolyticus* strains to form biofilms using the semi-quantitative adhesion test. The “Standardized biofilm” refers to the average value of 3 replicates; the “error bar” represents standard deviation of 3 replicates.

Semi-quantitative adhesion test revealed that the 47 strains exhibited diverse biofilm-forming abilities after 36 hrs of incubation (Figure 2). We further grouped the 47 strains based on their level of biofilm formation namely, no biofilm (12 strains; standardized biofilm: <0.01), weak biofilm producers (32 strains, standardized biofilm: 0.01–0.05), moderate producers (2 strains; standardized biofilm: 0.05–0.15), and a strain that had the highest biofilm production (standardized biofilm: >0.15).

Furthermore, among these strains two showed remarkably contrasting responses relative to 3-oxo-C_10_-HSL production (Table 2) and biofilm formation (Figure 2). Specifically, strain N°24 produced 3-oxo-C_10_-HSL with weak biofilm formation, while strain N°40 produced high levels of 3-oxo-C_10_-HSL accompanied by having the strongest biofilm formation. Thus, the contrasting characteristics of biofilm formation of these 2 strains could suggest the distinct strain specificity in the effects of exogenous 3-oxo-C_10_-HSL.

**Figure 3:**
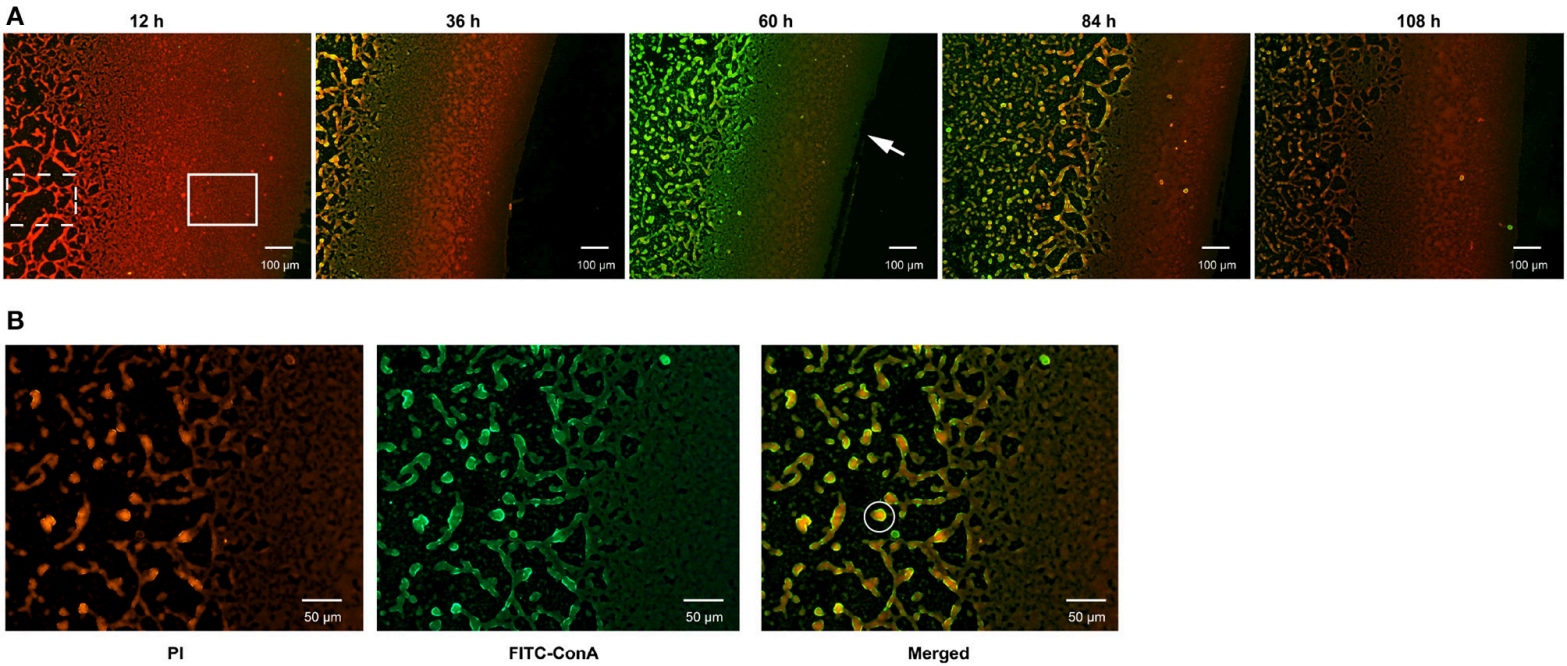
Determination of biofilm formation in *V. alginolyticus* by FLM. **(A)** Representative images of biofilm formed by strain N°40. Bar: 100 μm. **(B)** The matrix and bacterial cells in the biofilm formed by strain N°40. Bar: 50 μm. The “Solid rectangular box” symbol represents biofilm matrix constructed in sheets; the “dotted rectangular box” symbol represents biofilm matrix in a network; the “solid circle” symbol represents a bacterial community formed by bacterial cells and matrix; the “arrow” symbol represents the liquid-air interface.

During culturing from 12 to 120 h, strain N°24 did not exhibit any bacterial cell adhesion, while strain N°40 generated a compact biofilm matrix with attached bacterial cells (Figure 3A). In addition, the solid biofilm structures of strain N°40 became visible after 12 h, reaching a maximum in biovolume in between 60 and 72 h, followed by a collapse beginning at 84 h. Moreover, the density of adhered bacterial cells reached highest at the liquid-air interface, where the biofilm was thought to be constructed in sheets. The structure gradually changed into a cross-linked network away from the interface, where the bacterial cells continued to grow and aggregated (Figure 3B).

### Effects of exogenous 3-oxo-C_10_-HSL on biofilm formation

#### Biofilm formation under different temperatures

**Figure 4:**
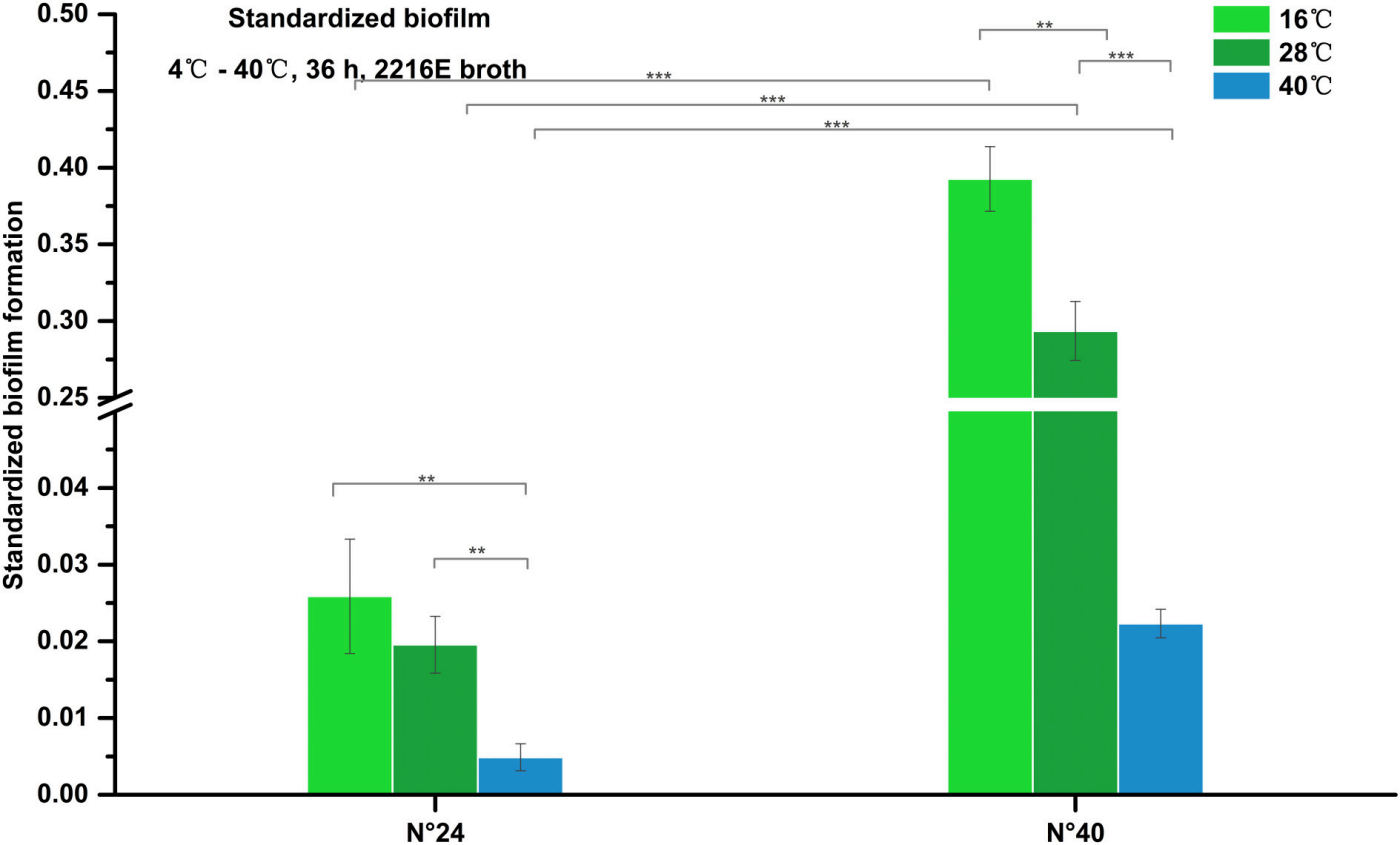
Biofilm formation of *V. alginolyticus* at different temperatures. Results of the semi-quantitative test of biofilm formation of strains N°24 and N°40 cultured for 36 h at 16, 28, and 40°C. The “Standardized biofilm” corresponds to the mean of 3 replicates with error bars for standard deviation; ^**^P < 0.01; ^***^P < 0.001.

As shown in Figure 4, biofilm formation of strain N°24 maintained very weak structure (standardized biofilm: 0.01–0.05) at 16°C and 28°C, and further decreased at 40°C (standardized biofilm: < 0.01). Biofilm formation of strain N°40 significantly increased at 16°C compared to that at 28°C but was significantly weaker (standardized biofilm: < 0.01) at 40°C. These indicate that to some extent, high temperature could inhibit *V. alginolyticus* biofilm formation.

#### Effects of 3-oxo-C_10_-HSL on biofilm formation at 16°C

**Figure 5:**
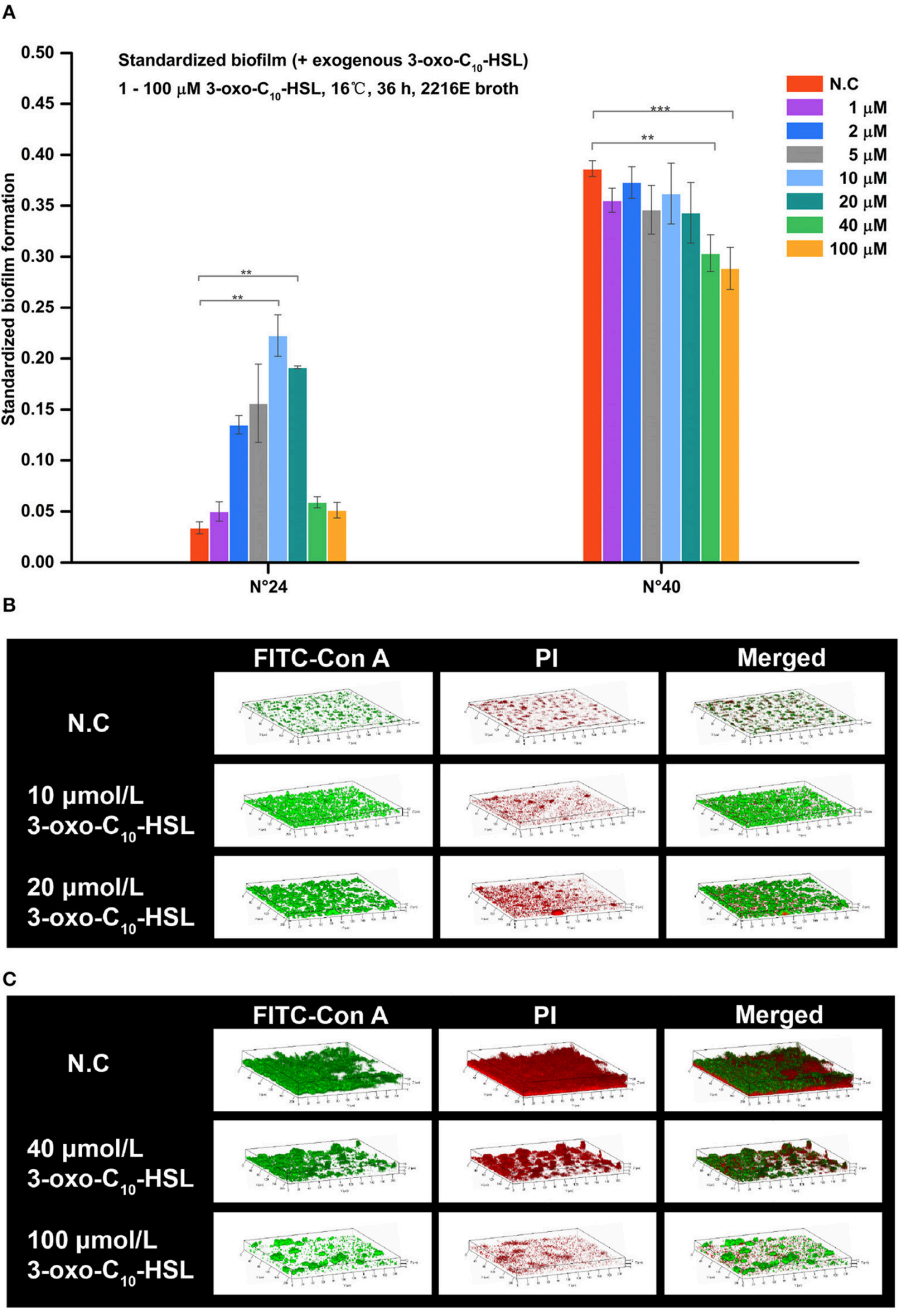
Biofilm formation of *V. alginolyticus* upon supplementation with exogenous 3-oxo-C_10_-HSL at 16°C. **(A)** Quantification of biofilms formed by strains N°24 and N°40 cultured for 36 h at 16°C using the semi-quantitative adhesion test. **(B)** Representative 3D-reconstructed biofilm structure of strain N°24 using CLSM. **(C)** Representative 3D-reconstructed biofilm structure of strain N°40 using CLSM. The “Standardized biofilm” corresponds to the average value of 3 replicates; the “error bar” represents standard deviation of 3 replicates; ^**^P < 0.01; ^***^P < 0.001; the “N.C” (negative control) refers to the strains cultured in 2216E broth without exogenous 3-oxo-C_10_-HSL for 36 h.

As shown in Figure 5A, the biofilms of strain N°24 significantly increased when supplemented with 2, 5, 10, and 20 μmol/L 3-oxo-C_10_-HSL. On the other hand, biofilm formation of strain N°40 significantly decreased when supplemented with 40 and 100 μmol/L 3-oxo-C_10_-HSL, but the other concentrations had no significant effects.

As shown in Figure 5B, strain N°24 formed a spotted biofilm in the negative control group but formed a sheet-like biofilm in groups supplemented with 10 and 20 μmol/L 3-oxo-C_10_-HSL. Biofilms of the 10 μmol/L 3-oxo-C_10_-HSL treated group were more homogeneous and thinner than the ridge-like biofilms of 20 μmol/L 3-oxo-C_10_-HSL treated group. As shown in Figure 5C, strain N°40 in the negative control group formed a thick but non-homogeneous biofilm, with bacterial cells covering the bottom of the matrix. However, in the 40 μmol/L 3-oxo-C_10_-HSL treated group, it formed a ridge-like and rougher biofilm with a significantly decreased matrix. Interestingly, in the 100 μmol/L 3-oxo-C_10_-HSL treated group, no biofilms were formed and only few colonies were observed.

Biofilms were thin and microcolonies were absent in the negative control of strain N°24. Compared to this, the biomass and roughness of the biofilms with added 10 and 20 μmol/L 3-oxo-C_10_-HSL were significantly enhanced, but the average thickness of both groups remained thin (5–7 μm) with several detected microcolonies. For strain N°40, the biomass and maximum thickness of the 40 and 100 μmol/L 3-oxo-C_10_-HSL treated groups decreased significantly compared to the negative controls, where microcolony counts decreased especially in the 100 μmol/L 3-oxo-C_10_-HSL but its roughness was enhanced at the same time (Table 3).

**Table 3:** Quantitative analysis of the biofilm matrices of *V. alginolyticus* strains at 16°C.

**Strains and culture conditions**		**BioMass (mg/cm^3^)**	**Average thickness (μm)**	**Max thickness (μm)**	**Roughness coefficient**	**Microcolonies at substrate**
N°24	N.C	0.550 ± 0.072	5.106 ± 1.103	10.000 ± 4.243	0.910 ± 0.090	ND
	10 μmol/L 3-oxo-C_10_-HSL	1.652 ± 1.198^*^	6.722 ± 1.725	17.000 ± 4.243^*^	1.543 ± 0.048^*^	2.500 ± 2.121^*^
	20 μmol/L 3-oxo-C_10_-HSL	1.543 ± 1.177^*^	5.438 ± 1.279	13.000 ± 1.414	1.189 ± 0.493^*^	1.500 ± 2.121^*^
N°40	N.C	13.588 ± 3.366	10.475 ± 3.763	26.000 ± 2.828	0.318 ± 0.060	50.000 ± 12.728
	40 μmol/L 3-oxo-C_10_-HSL	3.627 ± 0.408^**^	6.558 ± 1.190	19.667 ± 0.577^*^	0.683 ± 0.103	39.667 ± 3.215
	100 μmol/L 3-oxo-C_10_-HSL	0.777 ± 0.022^**^	7.580 ± 0.511	18.500 ± 2.121^*^	1.494 ± 0.022^*^	1.000 ± 0.000^*^

Biofilm matrix index values were analyzed using COMSTAT software and shown as mean of 3 replicates with standard deviation. The “N.C” (negative control) refers to the strains cultured in 2216E broth without exogenous 3-oxo-C_10_-HSL for 36 h; the “ND” - not detected;

* P < 0.05;

** *P < 0.01*.

#### Effects of 3-oxo-C_10_-HSL on biofilm formation at 28°C

**Figure 6:**
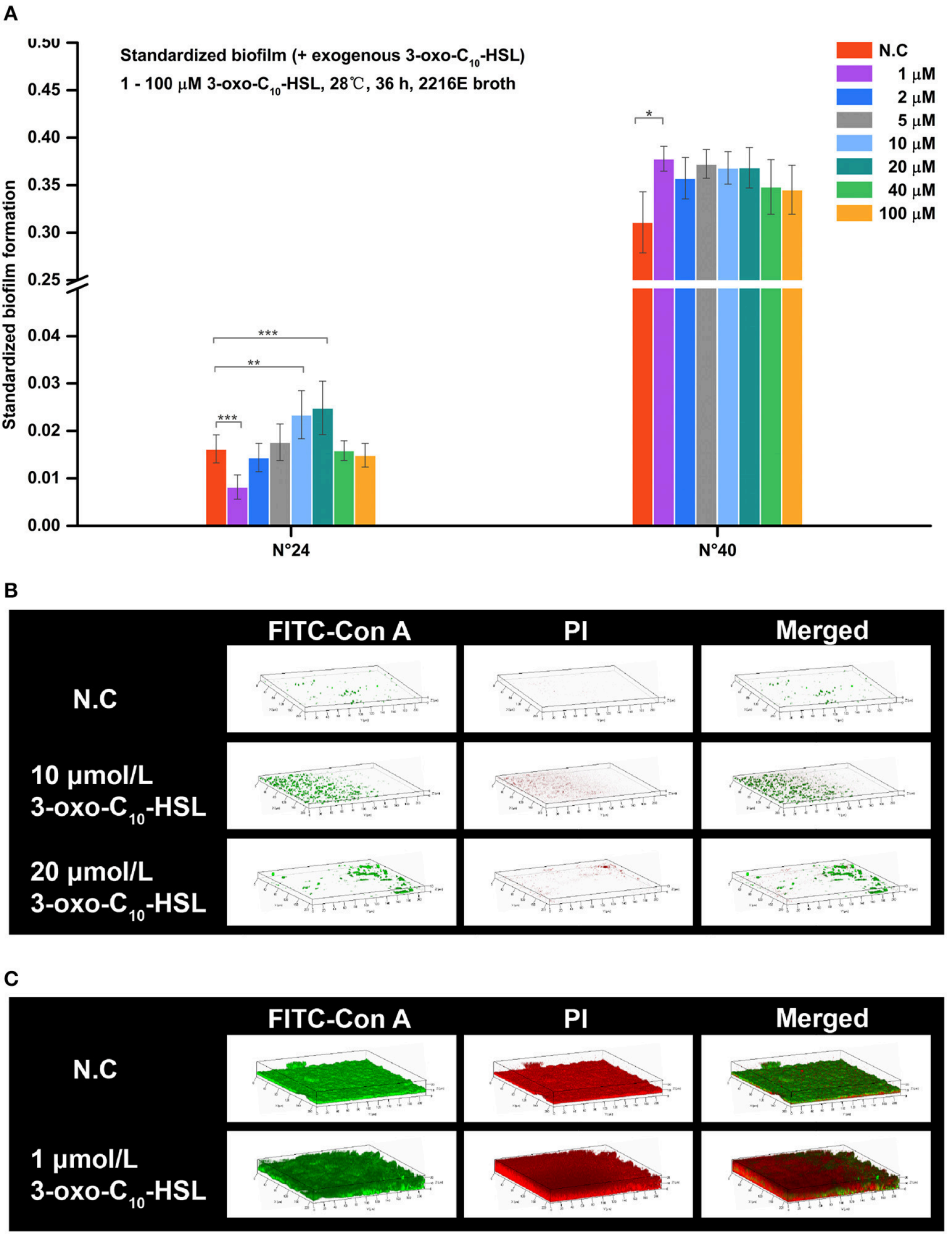
Biofilm formation of *V. alginolyticus* supplemented with exogenous 3-oxo-C_10_-HSL at 28°C (A) Quantification of biofilm formed by strains N°24 and N°40 cultured for 36 h at 28°C using the semi-quantitative adhesion test. **(B)** Representative 3D-reconstructed biofilm structure of strain N°24 using CLSM. **(C)** Representative 3D-reconstructed biofilm structure of strain N°40 using CLSM. The “Standardized biofilm” corresponds to the average value of 3 replicates; the “error bar” represents standard deviation of 3 replicates; ^*^P < 0.05; ^**^P < 0.01; ^***^P < 0.001; the “N.C” (negative control) refers to the strains cultured in 2216E broth without exogenous 3-oxo-C_10_-HSL for 36 h.

Addition of 3-oxo-C_10_-HSL also affected the formation of biofilms as shown in Figure 6A. For example, the biofilms of strain N°24 significantly decreased in 1 μmol/L treated group but became significantly higher in treatments added with 10 and 20 μmol/L. In contrast, biofilm formation in strain N°40 was only increased in 1 μmol/L 3-oxo-C_10_-HSL treated group.

Strain N°24 in the negative control group had pinpoint-like biofilm structure with no bacterial colonies (Figure 6B). However, sparse biofilms with adhered bacterial cells were detected in 10 μmol/L 3-oxo-C_10_-HSL treated group, and scattered biofilms with small colonies were also detected in 20 μmol/L 3-oxo-C_10_-HSL treated group. Meanwhile, strain N°40 in the negative control group formed a solid biofilm matrix with approximately 100% substratum coverage (Figure 6C). In 1 μmol/L 3-oxo-C_10_-HSL treated group, thicker biofilms with multiple layers of bacterial cells adhered to the matrix were observed.

For strain N°24, the biomass and roughness of the 10 and 20 μmol/L 3-oxo-C_10_-HSL treated groups were significantly enhanced compared to the negative control group. Its thickness also increased when added with 10 μmol/L of 3-oxo-C_10_-HSL but more microcolonies formed after addition of 20 μmol/L. For strain N°40, the roughness of the 1 μmol/L 3-oxo-C_10_-HSL treated group were the same as those in the negative control group, while the biomass and average thickness increased with more microcolonies (Table 4).

**Table 4:** Quantitative analysis of the biofilm matrices of *V. alginolyticus* strains at 28°C.

**Strains and culture conditions**		**BioMass (mg/cm^3^)**	**Average thickness (μm)**	**Max thickness (μm)**	**Roughness coefficient**	**Microcolonies at substrate**
N°24	N.C	0.046 ± 0.024	3.736 ± 1.247	8.500 ± 2.121	0.491 ± 0.358	2.500 ± 0.707
	1 μmol/L 3-oxo-C_10_-HSL	ND	ND	ND	ND	ND
	10 μmol/L 3-oxo-C_10_-HSL	2.949 ± 0.086^**^	6.090 ± 2.083^*^	9.000 ± 1.414	1.842 ± 0.138^**^	2.500 ± 0.707
	20 μmol/L 3-oxo-C_10_-HSL	3.273 ± 0.127^**^	4.090 ± 0.628	11.000 ± 1.414	1.297 ± 0.646^**^	6.000 ± 1.414
N°40	N.C	9.984 ± 1.677	12.987 ± 2.124	21.000 ± 2.646	0.187 ± 0.025	36.733 ± 4.162
	1 μmol/L 3-oxo-C_10_-HSL	15.167 ± 2.262^*^	17.808 ± 2.556^*^	21.500 ± 2.364^*^	0.234 ± 0.074	43.667 ± 3.786

Biofilm matrix index values were analyzed using COMSTAT software and shown as mean of 3 replicates with standard deviation. The “N.C” (negative control) refers to the strains cultured in 2216E broth without exogenous 3-oxo-C_10_-HSL for 36 h; the “ND” for not detected;

* P < 0.05;

** *P < 0.01*.

## Discussion

### AHL profiles of *V. alginolyticus*

As essential components of QS systems, AHLs have been detected in many *Vibrio* species, including *V. sinaloensis, V. brasiliensis, V. ichthyoenteri, V. vulnificus, V. scophthalmi, V. anguillarum* (Buchholtz et al., 2006; Garcia-Aljaro et al., 2008; Valiente et al., 2009; Li et al., 2010; Tan P. W. et al., 2014; Tan W. S. et al., 2014). However, to date, no systematic study of AHL distribution in *V. alginolyticus* are available, which hinders further understanding on the mechanisms underlying *V. alginolyticus* infection controls associated with QS system. Thus, the first step in our study was to classify and identify the wide range of AHLs produced by the *V. alginolyticus* isolates.

In this study, the negative results seen in *C. violaceum* CV026 detection suggest that the short side-chain AHLs might not be produced in *V. alginolyticus*, or that the AHL concentrations were below the detectable limit, consistent with those reported by Nievas et al. (2012) using the same detection assay. In contrast, the positive detection in *A. tumefaciens* KYC55 indicated that most tested *V. alginolyticus* strains could produce various AHLs (C_4_-HSL~C_14_-HSL), most of which could be long side-chain AHLs (C_8_-HSL~C_14_-HSL) with varying level of AHL production among strains. The HPLC-MS/MS we used to quantify the AHLs provided more detailed information on their production (Purohit et al., 2013). Out of the 14 target AHLs, our results confirmed 11 kinds of AHLs existing in *V. alginolyticus*, including both short and long side-chains, mainly dominated by 3-OH-C_4_-HSL, 3-oxo-C_10_-HSL, and 3-oxo-C_14_-HSL. Despite the exhaustive methods we used to profile the AHLs, the other types may also be produced by *V. alginolyticus* but not just detected in this study which warrants further studies and investigations.

Current studies reported a wide range of AHLs produced by *Vibrio* including C_4_-HSL, 3-OH-C_4_-HSL, C_6_-HSL, 3-oxo-C_6_-HSL, 3-OH-C_6_-HSL, C_8_-HSL, 3-OH-C_8_-HSL, C_10_-HSL, 3-OH-C_10_-HSL, 3-oxo-C_10_-HSL, 3-OH-C_12_-HSL, 3-oxo-C_12_-HSL (Buchholtz et al., 2006; Garcia-Aljaro et al., 2008; Valiente et al., 2009; Li et al., 2010; Purohit et al., 2013; Tan P. W. et al., 2014; Tan W. S. et al., 2014). In addition to these AHLs, we report for the first time C_12_-HSL and 3-oxo-C_14_-HSL for the genus *Vibrio*, suggesting a greater diversity of AHLs in the genus and this research area is worthy of further exploration.

### Biofilm formation of *V. alginolyticus*

In our study, only 6.4% *V. alginolyticus* isolates formed strong biofilms. Using similar biofilm detection method, our result was in contrast with the study of Snoussi et al. (2008), who found that 87.5% of their environmental *V. alginolyticus* isolates had strong biofilm forming abilities. This difference was most likely attributed to the origin of the isolates, culture medium and temperature, and further implies that biofilm formation in *V. alginolyticus* is greatly influenced by environmental conditions.

Indeed, our results revealed that temperature affected *V. alginolyticus* biofilm formation. Stronger biofilms were formed at 16°C than at 28°C, indicating higher biofilm biomass at relatively lower temperature. Studies on *V. cholerae* by Townsley and Yildiz (2015) also revealed a more compact biofilm matrix at lower temperature, consistent with our findings. In addition, biofilm structures became smoother with increasing temperature. Similar observations were reported by Remuzgo-Martinez et al. (2015). The rougher biofilms could provide larger surface area in favor of bacterial metabolism, while the smoother biofilms provide a stable environment to allow dormancy of bacterial cells (Donlan, 2002). Again, biofilm formation was completely inhibited at higher temperature (40°C), even though *V. alginolyticus* strains were still growing. This response was also found in the biofilm formation of other bacteria such as *Salmonella enteric* (Piras et al., 2015). The inhibition of *V. alginolyticus* biofilm formation at high temperature could be associated with the degradation of the AHLs involved in biofilm formation, resulting to the total inhibition of production of AHL-controlled EPS (Yates et al., 2002).

### Exogenous 3-oxo-C_10_-HSL affects biofilm formation in *V. alginolyticus*

Various physiological functions of long side-chain AHLs have been verified in many studies, such as biofilm formation (Nievas et al., 2012). Previous studies have confirmed that 3-oxo-C_10_-HSL was highly correlated to bacterial pathogenicity, and that it could regulate several bacterial biological processes such as in activating expression of virulence-related genes (Buchholtz et al., 2006; Wang et al., 2013). It also participates in the red tide occurrences by causing the microalgae *Ponticoccus* sp. to aggregate and form massive blooms (Chi et al., 2017).

We found that exogenous 3-oxo-C_10_-HSL differently affected *V. alginolyticus* biofilm formation, with apparent strain specificity. For a strain that did not produce 3-oxo-C_10_-HSL as that made weak biofilms, moderate concentration of 3-oxo-C_10_-HSL promoted biofilm formation, and the corresponding morphological changes included increased cell auto-aggregation and biofilm heterogeneity. The morphological changes provided a suitable condition for the adhesion of bacterial cells to the biofilm surface, which was also observed before. For example, Huang et al. (2009) found that the dominant AHLs changed from short-chain to long-chain AHLs during subtidal biofilm development, and this change provided a heterogeneous environment in favor of more distinct bacterial community development and even for biofilm formation. The same effects were reported by Nievas et al. (2012), where moderate addition of 3-oxo-C_10_-HSL (10 or 20 μmol/L) also increased biofilm formation of non-AHL-producing peanut-nodulating bacteria. For the strain with high level of 3-oxo-C_10_-HSL and strong biofilms, low concentration of 3-oxo-C_10_-HSL still promoted biofilm formation but further addition inhibited the activity. Decreased cell auto-aggregation and biofilm integrity were associated with this inhibition. We presumed that in AHL-producing *V. alginolyticus* strains, both exogenous and endogenous 3-oxo-C_10_-HSL had possible cumulative effects on biofilm formation. This hypothesis however needs further validation and verification. In addition, the effects of exogenous 3-oxo-C_10_-HSL on the same *V. alginolyticus* strain varied under different temperature regimes, implying that 3-oxo-C_10_-HSL effects could be influenced by changes in temperature.

## Conclusion

Our study characterized 11 different AHLs produced by 47 *V. alginolyticus* strains, and further explored the production and the effect of AHLs on the regulation of *V. alginolyticus* biofilm formation. We confirmed the presence of AHLs, and the dominant kinds of AHL signals produced by the 47 *V. alginolyticus* strains, and proposed a functional role of 3-oxo-C_10_-HSL on biofilm formation. We also showed that temperature played an apparent role in regulating the said processes. Our results provide new insights for future studies.

## Author contributions

KF and LZ conceptualized this study; JL, KF, YG, and LZ designed the research; JL, KF, YW, and CW conducted the experiments; JL, FL, and LS confirmed bacterial source and analyzed the data; JL and KF prepared the manuscript. JL and KF contributed equally to this work. All authors discussed the results, agreed on the interpretation and contributed in the finalization of the manuscript.

### Conflict of interest statement

The authors declare that the research was conducted in the absence of any commercial or financial relationships that could be construed as a potential conflict of interest.
