# Supplementary material for: Detection of Diverse N-Acyl-Homoserine Lactones in Vibrio alginolyticus and Regulation of Biofilm Formation by N-(3-Oxodecanoyl) Homoserine Lactone In vitro
Source: Front Microbiol. 2017 Jun 16;8:1097. doi: 10.3389/fmicb.2017.01097 (PMC5472671; doi:10.3389/fmicb.2017.01097)
Supplement: Supplementary file 1 [file Table1.DOCX]

Supplementary Material

**Detection of diverse N-acyl-homoserine lactones in *Vibrio alginolyticus* and regulation of biofilm formation by N-(3-oxodecanoyl) homoserine lactone in vitro**

Jianfei Liu^¶^, Kaifei Fu^¶^, Yuxiao Wang, Chenglin Wu, Fei Li, Lei Shi, Yanjun Li, Yinlin Ge^*^, Lijun Zhou^*^

**^¶^Authors contributed equally to this work.**

***Correspondence:** Dr. Yinlin Ge: [geyinlin@126.com](mailto:geyinlin@126.com); Dr. Lijun Zhou: [hzzhoulj@126.com](mailto:hzzhoulj@126.com)

**Supplementary Table 1 | Bacterial strains.**

| Strains | Description | Source |
| --- | --- | --- |
| *V. alginolyticus* N°01 - N°47 | marine-isolated *V. alginolyticus* strains | This study |
| *C. violaceum* CV026 | Biosensor strain for short side-chain AHLs | McClean KH *et al*. (1997) |
| *A. tumefaciens* KYC55 (pJZ372)(pJZ384)(pJZ410) | Biosensor strain for long side-chain AHLs | Zhu *et al.* (2003) |
| *E. carotovora* GS101 | Positive strain for short side-chain AHLs detection | Chhabra SR *et al.* (1993) |
| *P. aeruginosa* PAO1 | Positive strain for long side-chain AHLs detection | Tateda K *et al.* (2003) |
