# Supplementary material for: Detection of Diverse N-Acyl-Homoserine Lactones in Vibrio alginolyticus and Regulation of Biofilm Formation by N-(3-Oxodecanoyl) Homoserine Lactone In vitro
Source: Front Microbiol. 2017 Jun 16;8:1097. doi: 10.3389/fmicb.2017.01097 (PMC5472671; doi:10.3389/fmicb.2017.01097)
Supplement: Supplementary file 2 [file Table2.DOCX]

Supplementary Material

**Detection of diverse N-acyl-homoserine lactones in *Vibrio alginolyticus* and regulation of biofilm formation by N-(3-oxodecanoyl) homoserine lactone in vitro**

Jianfei Liu^¶^, Kaifei Fu^¶^, Yuxiao Wang, Chenglin Wu, Fei Li, Lei Shi, Yanjun Li, Yinlin Ge^*^, Lijun Zhou^*^

**^¶^Authors contributed equally to this work.**

***Correspondence:** Dr. Yinlin Ge: [geyinlin@126.com](mailto:geyinlin@126.com); Dr. Lijun Zhou: [hzzhoulj@126.com](mailto:hzzhoulj@126.com)

**Supplementary Table 2 | Linear gradient elution condition of HPLC.**

| Time  (min) | Mobile phase A  (0.1% v/v formic acid and 2 mM ammonium acetate in water) | Mobile phase B  (0.1% v/v formic acid and 2 mM ammonium acetate in methanol) |
| --- | --- | --- |
| 0.1 | 70% | 30% |
| 8 | 50% | 50% |
| 24 | 10% | 90% |
| 32 | 0% | 100% |
| 36 | 0% | 100% |
| 36.1 | 70% | 30% |
| 40 | 70% | 30% |
| 40.1 | Stop | Stop |
