# Supplementary material for: Detection of Diverse N-Acyl-Homoserine Lactones in Vibrio alginolyticus and Regulation of Biofilm Formation by N-(3-Oxodecanoyl) Homoserine Lactone In vitro
Source: Front Microbiol. 2017 Jun 16;8:1097. doi: 10.3389/fmicb.2017.01097 (PMC5472671; doi:10.3389/fmicb.2017.01097)
Supplement: Supplementary file 3 [file Table3.DOCX]

Supplementary Material

**Detection of diverse N-acyl-homoserine lactones in *Vibrio alginolyticus* and regulation of biofilm formation by N-(3-oxodecanoyl) homoserine lactone in vitro**

Jianfei Liu^¶^, Kaifei Fu^¶^, Yuxiao Wang, Chenglin Wu, Fei Li, Lei Shi, Yanjun Li, Yinlin Ge^*^, Lijun Zhou^*^

**^¶^Authors contributed equally to this work.**

***Correspondence:** Dr. Yinlin Ge: [geyinlin@126.com](mailto:geyinlin@126.com); Dr. Lijun Zhou: [hzzhoulj@126.com](mailto:hzzhoulj@126.com)

**Supplementary Table 3 | MRM mode conditions of 14 AHL standards.**

| Standard compound | Molecular formula | Molecular weight | *m/z*  *(*Da) | Peak time  (min) | DP  (volts) | EP  (volts) | CE  (volts) | CXP  (volts) |
| --- | --- | --- | --- | --- | --- | --- | --- | --- |
| C_4_-HSL | C_8_H_13_NO_3_ | 171.19 | 172.099/71.000 | 1.49 | 56 | 10 | 15 | 6 |
| 3-OH-C_4_-HSL | C_8_H_13_NO_4_ | 187.19 | 188.688/81.900 | 1.38 | 116 | 10 | 35 | 10 |
| C_6_-HSL | C_10_H_17_NO_3_ | 199.25 | 200.169/102.000 | 7.35 | 96 | 10 | 15 | 14 |
| 3-oxo-C_6_-HSL | C_10_H_15_NO_4_ | 213.23 | 214.116/101.900 | 1.46 | 96 | 10 | 15 | 16 |
| C_8_-HSL | C_12_H_21_NO_3_ | 227.3 | 228.086/127.000 | 14.70 | 76 | 10 | 15 | 14 |
| 3-OH-C_8_-HSL | C_12_H_21_NO_4_ | 243.30 | 244.241/226.200 | 24.40 | 86 | 10 | 13 | 10 |
| 3-oxo-C_8_-HSL | C_12_H_19_NO_4_ | 241.28 | 242.065/102.100 | 8.99 | 81 | 10 | 15 | 8 |
| C_10_-HSL | C_14_H_25_NO_3_ | 255.35 | 256.228/102.000 | 20.90 | 206 | 10 | 27 | 14 |
| 3-oxo-C_10_-HSL | C_14_H_23_NO_4_ | 269.34 | 270.208/88.000 | 21.30 | 261 | 10 | 29 | 10 |
| C_12_-HSL | C_16_H_29_NO_3_ | 283.41 | 284.274/102.000 | 24.10 | 211 | 10 | 29 | 14 |
| 3-OH-C_12_-HSL | C_16_H_29_NO_4_ | 299.41 | 300.099/283.100 | 21.00 | 46 | 10 | 13 | 14 |
| 3-oxo-C_12_-HSL | C_16_H_27_NO_4_ | 297.39 | 298.228/102.100 | 22.10 | 291 | 10 | 31 | 12 |
| 3-OH-C_14_-HSL | C_18_H_33_NO_4_ | 327.46 | 328.255/102.100 | 24.2 | 96 | 10 | 17 | 16 |
| 3-oxo-C_14_-HSL | C_18_H_31_NO_4_ | 325.44 | 326.197/144.000 | 24.40 | 76 | 10 | 21 | 20 |

Product ions with underline were quantitation ions.
