# Supplementary material for: Detection of Diverse N-Acyl-Homoserine Lactones in Vibrio alginolyticus and Regulation of Biofilm Formation by N-(3-Oxodecanoyl) Homoserine Lactone In vitro
Source: Front Microbiol. 2017 Jun 16;8:1097. doi: 10.3389/fmicb.2017.01097 (PMC5472671; doi:10.3389/fmicb.2017.01097)
Supplement: Supplementary file 4 [file Table4.DOCX]

Supplementary Material

**Detection of diverse N-acyl-homoserine lactones in *Vibrio alginolyticus* and regulation of biofilm formation by N-(3-oxodecanoyl) homoserine lactone in vitro**

Jianfei Liu^¶^, Kaifei Fu^¶^, Yuxiao Wang, Chenglin Wu, Fei Li, Lei Shi, Yanjun Li, Yinlin Ge^*^, Lijun Zhou^*^

**^¶^Authors contributed equally to this work.**

***Correspondence:** Dr. Yinlin Ge: [geyinlin@126.com](mailto:geyinlin@126.com); Dr. Lijun Zhou: [hzzhoulj@126.com](mailto:hzzhoulj@126.com)

**Supplementary Table 4 | Linear equation and correlation coefficient of AHL standards.**

| Standard compound | Linear regression equation | Correlation coefficient (*r)* |
| --- | --- | --- |
| C_4_-HSL | Y=6.4*10^3^ x + 2.75*10^3^ | 0.9988 |
| 3-OH-C_4_-HSL | Y=1.04 x + 570 | 0.9956 |
| C_6_-HSL | Y=3.5*10^3^ x + 714 | 0.9980 |
| 3-oxo-C_6_-HSL | Y=1.03*10^4^ x + 757 | 0.9996 |
| C_8_-HSL | Y=2.25*10^4^ x + 2.46*10^3^ | 0.9921 |
| 3-OH-C_8_-HSL | Y=3.18*10^3^ x + 1.84*10^3^ | 0.9985 |
| 3-oxo-C_8_-HSL | Y=1.36*10^4^ x + 2.26*10^3^ | 0.9980 |
| C_10_-HSL | Y=80.8x + 369 | 0.9954 |
| 3-oxo-C_10_-HSL | Y=3.77 x + 138 | 0.9946 |
| C_12_-HSL | Y=148 x + 634 | 0.9955 |
| 3-OH-C_12_-HSL | Y=2.56*10^3^ x + 6.09*10^3^ | 0.9979 |
| 3-oxo-C_12_-HSL | Y=105x + 85 | 0.9904 |
| 3-OH-C_14_-HSL | Y=9.2*10^4^ x + 2.03*10^4^ | 0.9995 |
| 3-oxo-C_14_-HSL | Y=45.5x + 2.14*10^3^ | 0.9994 |
